# Single-cell transcriptome profiling highlights the role of APP in blood vessels in assessing the risk of patients with proliferative diabetic retinopathy developing Alzheimer’s disease

**DOI:** 10.3389/fcell.2023.1328979

**Published:** 2024-01-24

**Authors:** Xiaoyu Xu, Cheng Zhang, Guoyi Tang, Ning Wang, Yibin Feng

**Affiliations:** School of Chinese Medicine, Li Ka Shing Faculty of Medicine, The University of Hong Kong, Hong Kong, China

**Keywords:** proliferative diabetic retinopathy, Alzheimer’s disease, vasculature, amyloid-beta precursor protein, endothelial cells

## Abstract

**Introduction:** The incidence of diabetic retinopathy (DR) has been found to be associated with the risk of developing Alzheimer‘s disease (AD). In addition to the common properties of neurodegeneration, their progressions are involved with abnormal vascular functions. However, the interactions between them have not been fully understood. This study aimed to investigate the key factor for the underlying interactions and shared signaling pathways in the vasculature of DR and AD.

**Methods:** We retrieved single-cell RNA sequencing (scRNA-seq) data regarding human fibrovascular membrane (FVM) of proliferative diabetic retinopathy (PDR) and human hippocampus vessels of AD from the NCBI-GEO database. GSEA analysis was performed to analyze AD-related genes in endothelial cells and pericytes of PDR. CellChat was used for predicting cell-cell communication and the signaling pathway.

**Results:** The data suggested that amyloid-beta precursor protein (APP) signaling was found crucial in the vasculature of PDR and AD. Endothelial cells and pericytes could pose influences on other cells mainly via APP signaling in PDR. The endothelial cells were mainly coordinated with macrophages in the hippocampus vasculature of AD via APP signaling. The bulk RNA-seq in mice with PDR validated that the expression of APP gene had a significant correlation with that of the AD genome-wide association studies (GWAS) gene.

**Discussion:** Our study demonstrates that the vasculopathy of PDR and AD is likely to share a common signaling pathway, of which the APP-related pathway is a potential target.

## Introduction

Diabetic retinopathy (DR) is a major diabetic complication that could induce severe vision impairment in patients with diabetes, and it has become a global health concern (Yau et al., 2012). Proliferative diabetic retinopathy (PDR) is the advanced stage of DR and is characterized by microvasculopathy, which can result in the formation of the fibrovascular membrane (FVM). The aberrant growth of leaky and fragile vessels in the FVM could easily lead to hemorrhage in vitreous and even retinal traction (Tamaki et al., 2016; Nawaz et al., 2019). Therefore, the pathology of FVM is important for the vasculopathy of PDR.

Alzheimer’s disease (AD) is a progressive neurodegenerative disease and attributes to 60%–70% of dementia cases, threatening the health of older adults worldwide (WHO, 2023). It is characterized by cognitive and memory impairment, and aging and vascular risk factors are the primary risk factors of AD (Hou et al., 2019). Its pathology is related to the amyloid plaques and neurofibrillary tangles, which can result in cerebral neurodegeneration (Lane et al., 2018). The brain vasculature is of great medical importance for brain health due to the special structure of the blood–brain barrier (BBB), which maintains the balance of molecule movement and interactions (Govindpani et al., 2019; Hussain et al., 2021). Abnormal vascular pathologies have been found in the aging brain and in AD, such as cerebral amyloid angiopathy and small vessel disease (Attems and Jellinger, 2014). Brain vascular dysfunction is indicated to promote the progression of AD.

**FIGURE 1:**
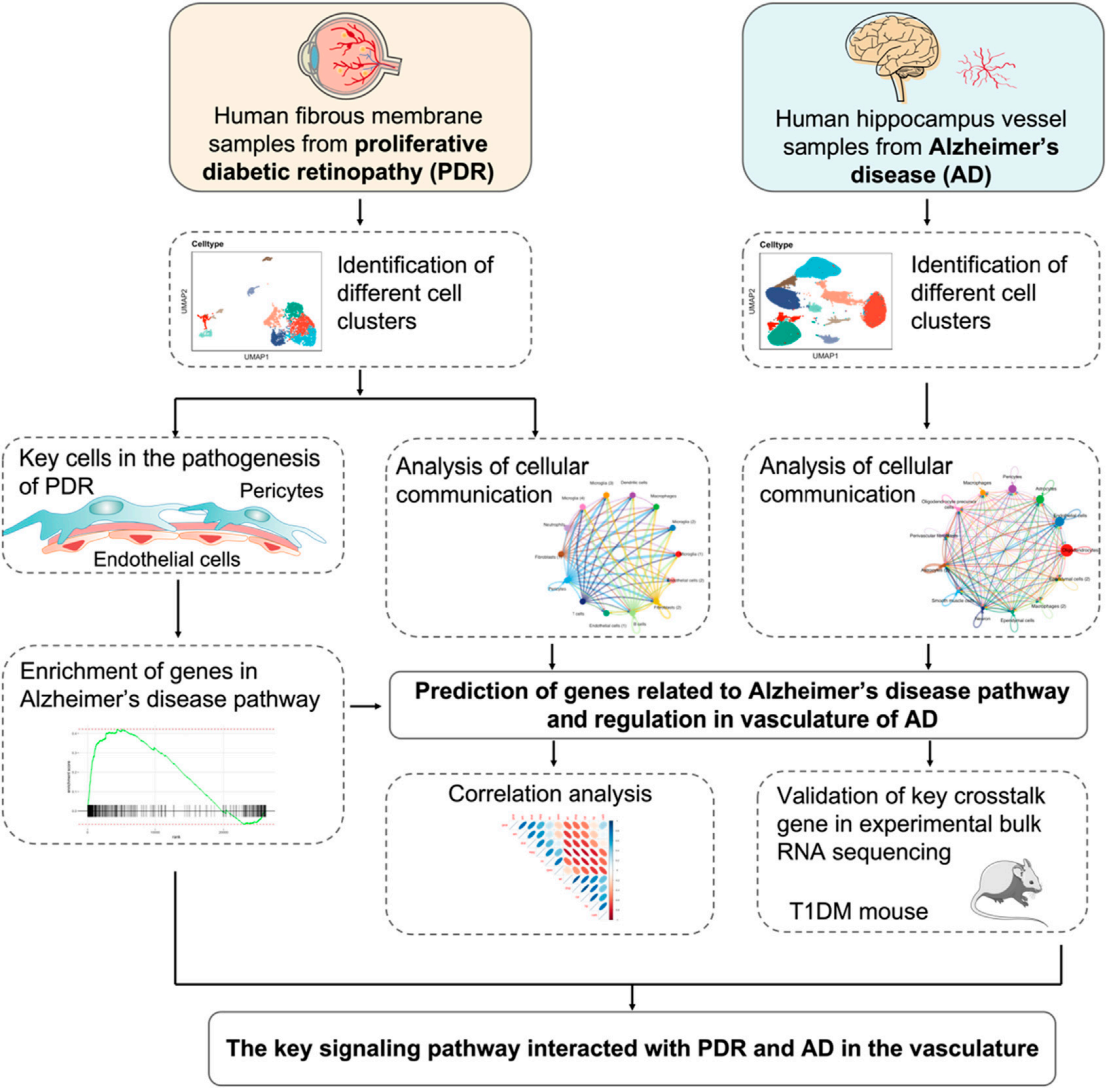
Schematics of the workflow of the whole study. The endothelial cell and pericyte clusters were selected from scRNA-seq of PDR and analyzed by GSEA KEGG enrichments. The cellular communication of PDR scRNA-seq was integrated to predict the genes related to AD pathway and elucidate its potential role. The snRNA-seq of AD vasculature was used to investigate cellular communication and further confirm the role of the predicted genes in the vasculature of AD. The predicted genes were validated by correlating with GWAS AD risk genes in the data sets of bulk RNA-seq from animal experiments. The key genes and potential signaling pathways involved with the vasculature of PDR were identified.

Recent studies have found that the incidence of DR correlates with brain neurodegenerative diseases like AD, and both these are involved with neurodegeneration and abnormal vascular functioning during development (Chua et al., 2022). A cohort study that included 134,327 diabetic patients above 60 years of age demonstrated that there was a higher percentage of patients with DR developing AD than diabetic patients without DR (Pedersen et al., 2022). Furthermore, the clinical findings reported that the deposition of retinal amyloid was higher in AD than in control subjects, and retinal vessel oscillations were altered in AD patients at the stage of dementia or mild cognitive impairment (Tadokoro et al., 2021; Kotliar et al., 2022). In addition, DR and AD were found to exhibit shared common pathogenic pathways and vascular regulatory properties in the microvasculature (Patton et al., 2005; Sundstrom et al., 2018). Therefore, the identification of DR by measuring certain biomarkers and ophthalmologic testing was considered a feasible way of screening and treating patients at risk of AD in the early stage (Martinez and Peplow, 2019; Grauslund et al., 2021). However, the interactions and common pathways in the vasculature of PDR and AD have not been clearly known. To address these questions, we combined the single-cell RNA sequencing (scRNA-seq) data sets from the vasculature of PDR and AD to gain further insights (Figure 1). Specifically, we conducted the analysis of scRNA-seq in FVM samples from PDR patients and selected the endothelial cell and pericyte clusters for GSEA Kyoto Encyclopedia of Genes and Genomes (KEGG) enrichment. CellChat was also carried out to highlight the key genes contributing to cellular communication for retinal endothelial cells and pericytes in PDR data sets. Integration of these results enabled us to identify the potential genes related to progression of AD in the retinal vasculature and characterize its regulatory role in the cell–cell communication. The role of key genes is further confirmed in the analysis of AD scRNA-seq data sets and the cellular communication in hippocampus blood vessels of AD. Lastly, by correlating the predicted genes with GWAS AD risk genes in scRNA-seq of PDR, snRNA-seq of AD, and the bulk RNA in the animal experiment data sets, we identified the key genes and potential signaling pathways that interacted with PDR and AD in the vasculature.

In the present study, we found that the amyloid-beta precursor protein (APP) signaling pathway was the main pathway responsible for the interactions in the vasculature between PDR and AD. The gene expression of *APP* was validated and correlated with the GWAS AD risk genes by the original bulk RNA-seq data of PDR. Hence, the APP signaling pathway may be a potential target for investigating the interactions in the vasculature of PDR and AD.

## Materials and methods

### Data acquisition and pre-processing

Human FVM of PDR (GSE 165784) and human hippocampus vasculature of AD (GSE 163577) snRNA-seq data were downloaded from the NCBI-GEO database. FVM samples were collected from two patients with PDR without any treatment of preoperative anti-VEGF or steroid injections in GSE 165784. The cells in the PDR data set were filtered with the quality control (nCount RNA >500, nFeature_RNA <5,000, and percent.mt <30), and 26,224 genes were used for the analysis. In addition, vessel samples isolated from the hippocampus of nine patients with AD were used in GSE 163577, and the nuclei in the AD data set were filtered with the quality control (nCount_RNA >200, nFeature_RNA <7000, and percent.mt <5), and 27,941 genes were used for the analysis. Data pre-processing, normalization, scaling, and cell clustering were performed with the Seurat package (version 4.3.0) in R (version 4.2.2). Cell clustering was determined by Seurat’s FindNeighbors, FindClusters, and RunUMAP functions. The marker genes were identified by the positive differential expression of each distinct cluster against all other clusters. The cell types were annotated based on the original results of the corresponding published articles and PanglaoDB (Hu et al., 2022; Yang et al., 2022).

The total RNA transcriptomic data of five human retina samples of PDR patients and 20 healthy people (GSE 160306) were downloaded from the NCBI-GEO database. The retina samples of PDR donors were obtained from donors diagnosed with PDR and diabetic macular edema (DME). The differentially expressed genes were obtained by using limma packages in R, and the cutoff to determine the significance was *p*-value <0.05.

### Gene set enrichment analysis

The gene set enrichment analysis (GSEA) calculates the enrichment score of the specified gene sets in different functional categories to find out the significant and concordant differences between two functional phenotypes (Joly et al., 2020). We performed GSEA in all genes of each cell cluster by using the Kyoto Encyclopedia of Genes and Genomes (KEGG) pathways database.

### Cell–cell communication

The Seurat-preprocessed data were further analyzed by using CellChat (version 1.6.1) to investigate the cell–cell interactions based on the expression of known ligand–receptor pairs in different cell types. We followed the official workflow and used the standard parameters set in the functions identifyOverExpressedGenes, identifyOverExpressedInteractions, and projectData to prepare the data set. The cell–cell contact subset of CellChatDB was used for the cell–cell communication analysis, and then we applied the core official functions and performed the visualization (Jin et al., 2021).

### Monocle trajectory analysis

Monocle was used to generate the pseudotime trajectory analysis in cell clusters of PDR and AD. The cells were preprocessed for clustering in Seurat and transferred into Moncole 3 and then used as input to perform trajectory graph learning and pseudotime measurements. The graph_test function was performed to calculate the value of morans_I of genes, and the tendency of gene expression in pseudotime was plotted by using plot_pseudotime_heatmap function.

### Average gene expression and correlation analysis

The averaged expression values of the selected genes were calculated by the AverageExpression function in the Seurat package for each cell cluster. The averaged expression values of genes were used for the correlation analysis. The correlation analysis was performed in the Cor function with the Spearman’s method for human PDR and AD data sets and the Pearson’s method for mouse PDR data sets. The significance of correlation was calculated by the cor_pmat function in the ggcorrplot package, and the visualization was completed by using corrplot, ggplot2, and ggcorrplot.

### Type I diabetic animal model

All animal protocols in this study were approved by the Committee on the Use of Live Animals in Teaching and Research of the University of Hong Kong. C57BL/6 mice aged 6–8 weeks were intraperitoneally injected with streptozotocin (STZ) at the dose of 55 mg/kg for 5 consecutive days to develop type I diabetes. After the 5-day injections, the mice with the level of fasting blood glucose over 15 mmol/L were included in the experiment. We provided the mice *ad libitum* access to food and water, and the feeding lasted for 14 weeks. At the end of the experiment, the level of fasting blood glucose was detected, and after deep anesthesia, the eyes of the mice were collected and the retina was isolated from the eyeballs for trypsin digestion and bulk RNA sequencing.

### Fundus photography

Fundus photography is used for measuring the severity of retinopathy in living mice. It was performed by the protocol of a previous published study (Zhang et al., 2021). Briefly, the ocular fundus was captured by an ophthalmoscope-dependent system equipped with a 78D ophthalmoscope on a smart phone and the application of Filmic Pro. Photography was conducted in a dark environment, and the lens was vertically placed above the cornea. We then adjusted the distance for focus and captured the photographs with the Filmic Pro App.

### Retinal permeability assessment

Evans blue dye (Sigma, United States, dissolved in 0.9% normal saline) was used to measure the integrity of the blood–retinal barrier according to the modified method (Wang et al., 2015; Zhang et al., 2021). Before the measurement, the mice were anesthetized, and then, the mice received intravenous injection with 45 mg/kg Evans blue dye. After the dye circulated in the system for 3 h, the mice were completely perfused by 0.1 M citrate buffer (pH = 3.5), the retina was immediately isolated, and the blood was collected. The retina was dried at 60°C for 5 h by using the CentriVap centrifugal vaccum concentrator (Labconco, United States), and the dried weight of the retina was recorded. The residue was placed in formamide at 70°C for 18 h to extract the Evans blue dye and centrifugated at 14,000 rpm at 4°C for 90 min. The absorbance of the supernatant and serum was measured at 620 nm. The concentration of the Evans blue dye was calculated by using a standard curve, and the blood–retinal barrier permeability was expressed as µL plasma × g retinal dry wt^−1^ h^−1^.

### Immunofluorescence of vasculature in the retina

After being isolated from the eyeballs, the retina was further fixed in 4% paraformaldehyde and cut into a flat-mounted form. It was then treated with 70% ethanol for 1 h, 1% Triton X-100 for 1 h, and the mixture of 10% goat serum and 0.3% Triton X-100 for 2 h for blocking. Afterward, the retina was immunostained with Alexa Fluor 488–conjugated Isolectin GS-IB4 (0.02 mg/mL) for 24 h. The images were captured with a confocal laser scanning microscope.

### Isolation of retinal vasculature by trypsin digestion

The retina was isolated from the eyeballs of the different groups and fixed with 4% paraformaldehyde. The fixed retina was incubated with 3% trypsin in 0.1 M Tris buffer (pH = 8.2) and was gently shaken for 3 h at 37°C. The digested tissues were washed away by PBS several times to isolate the retinal vasculature, and it was further stained with H&E to analyze the retinal vessels that included endothelial cells, pericytes, and acellular capillaries.

## Results

### Identification of highly expressed gene candidates in endothelial cells and pericytes in PDR and AD

**FIGURE 2:**
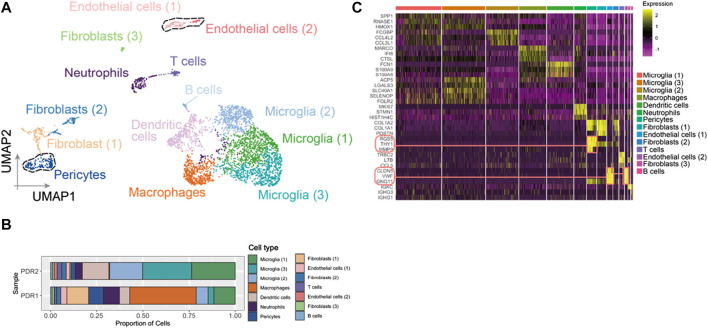
Endothelial cells and pericytes were identified in FVM of PDR. **(A)** UMAP displaying a total of 14 cell clusters in FVM samples. There were four microglia clusters, two fibroblast clusters, two endothelial cell clusters, one dendritic cell cluster, one cluster as pericytes, one cluster as neutrophils, one cluster as macrophages, one cluster as T cells, and one cluster as B cells. **(B)** Bar plot showing the distribution of cell type proportions across FVM samples from PDR patients based on scRNA-seq. Each cell type is represented by a distinct color as shown in **(A)**. The number of cells in each cell type per donor is listed in Supplementary Table S1. **(C)** Heatmap of top three marker genes in each cluster. The marker genes in endothelial cell and pericyte clusters are highlighted.

After the quality control filtering, we obtained 3,584 single cells from FVM samples of PDR for further analysis. We performed UMAP for cell clustering and obtained 14 cell clusters: four clusters as microglia, two clusters as fibroblasts, two clusters as endothelial cells, one cluster as dendritic cells, one cluster as pericytes, one cluster as neutrophils, one cluster as macrophages, one cluster as T cells, and one cluster as B cells in FVM of PDR (Figure 2A). The distributions of cell type proportions across the samples showed that the endothelial cells and pericytes accounted for 10%–20% of the total (Figure 2B; Supplementary Table S1) in FVM of PDR patients, which played an important role in the vasculature progression of PDR. We selected endothelial cells and pericytes in PDR for further analysis and investigated the expression pattern of marker genes in each cell cluster by constructing a heatmap (Figure 2C). We found that both clusters of endothelial cells (1) and (2) were enriched in the endothelial cell maker genes *GNG11*, *VWF*, and *CLDN5*. The cluster of pericytes expressed a high level of the marker genes *MMP9*, *RGS5*, and *THY1*.

### Retinal vasculature in PDR had an association with the neurodegenerative diseases

**FIGURE 3:**
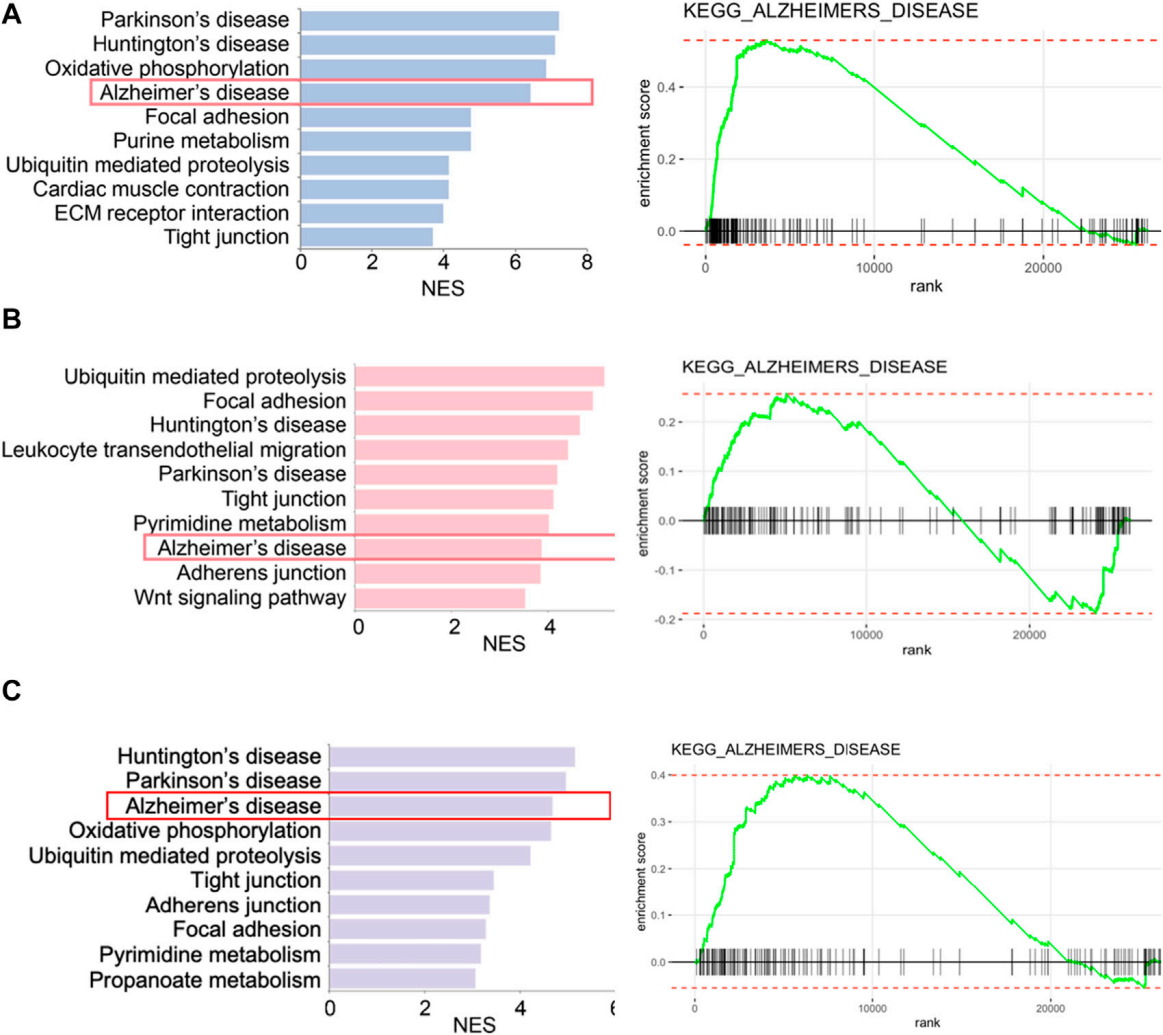
Enriched biological pathways by GSEA in two endothelial cell clusters and one pericyte cluster from FVM of PDR. **(A)** Left, representative enriched biological pathway in KEGG in the pericytes cluster. Right, GSEA enrichment plot of the AD pathway in the pericytes cluster. **(B)** Left, representative enriched biological pathway in KEGG in the endothelial cells (1) cluster. Right, GSEA enrichment plot of the AD pathway in the endothelial cells (1) cluster. **(C)** Left, representative enriched biological pathway in KEGG in the endothelial cells (2) cluster. Right, GSEA enrichment plot of the AD pathway in the endothelial cells (2) cluster. NES, normalized enrichment score. All displayed pathways had *p*-value < 0.05.

**FIGURE 4:**
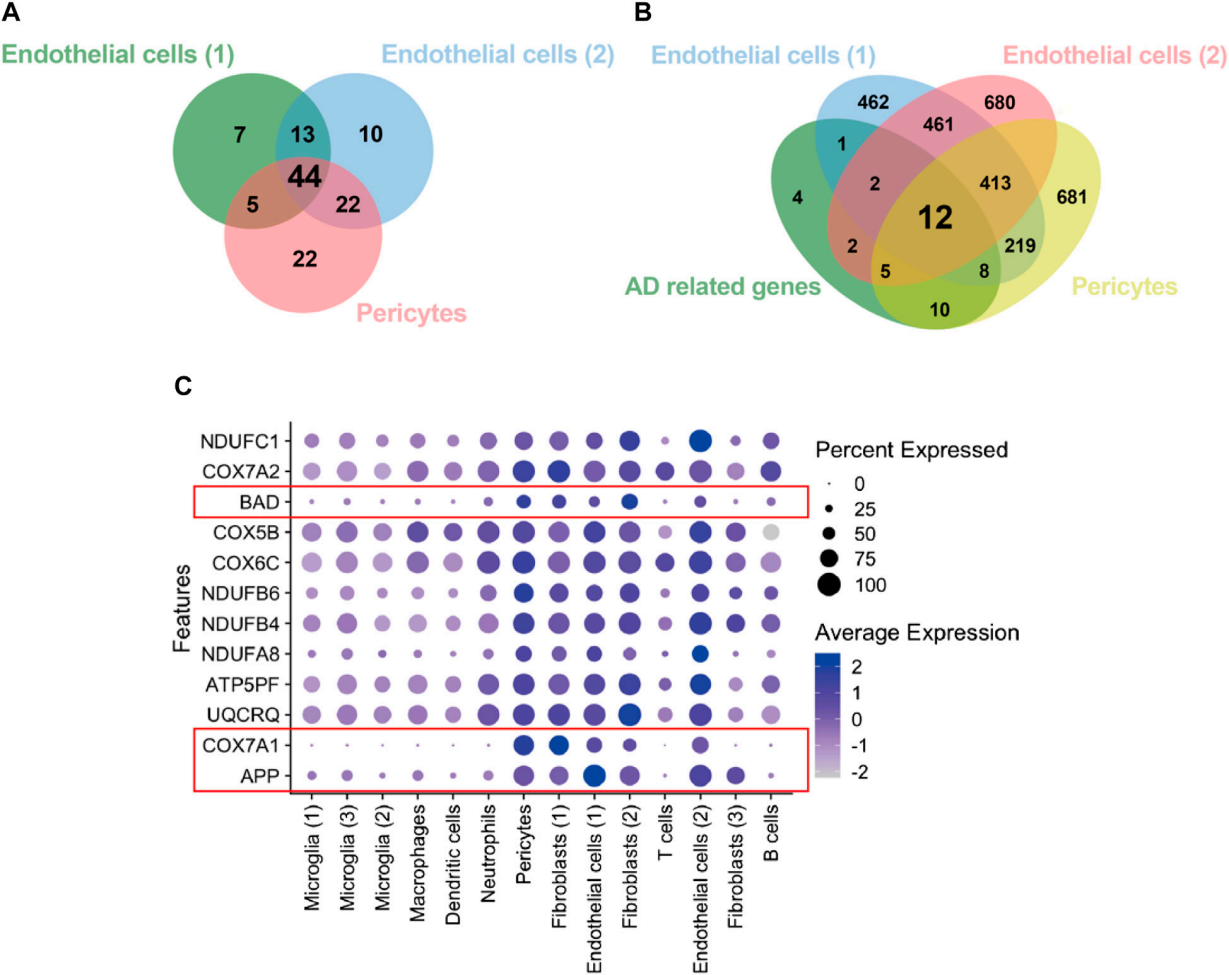
Identification of genes from the endothelial cells and pericytes in PDR related to the AD pathway. **(A)** Integration of KEGG enrichment genes in the AD pathway from endothelial cells and pericytes in PDR. **(B)** Integration of highly expressed genes in both endothelial cells and pericytes with AD pathway–related genes. AD-related genes: 44 overlapping genes **(A)**. **(C)** Feature plot of 12 AD-related genes with high expression in endothelial cell and pericyte clusters.

We retrieved the gene expression data from two endothelial cell clusters and a pericyte cluster, encompassing a total of 26,244 genes, to identify key genes by the GSEA with KEGG pathway data sets. The results showed high enrichment scores in pathways of neurodegenerative diseases like AD, Parkinson’s disease (PD), and Huntington’s disease (HD). The AD pathway was significantly enriched in both endothelial cell and pericyte clusters (Figure 3; Supplementary Tables S2–S4). The Venn diagram showed that among these genes in the AD pathway, 44 genes were overlapped in three clusters (Figure 4A; Supplementary Table S5), which indicated that the endothelial cells and pericytes from PDR might have association with the development of AD. To find out which AD-related genes were specifically expressed in the endothelial cells and pericytes, genes that were identified as the marker genes in both the endothelial cell and pericyte clusters with differentially upregulated expression by the FindAllMarkers function were overlapped with the genes enriched in the AD pathway. A total of 12 genes related to the AD pathway were highly expressed in the endothelial cells and pericytes, which included *APP*, *COX7A1*, *UQCRQ*, *ATP5PF*, *NDUFA8*, *NDUFB4*, *NDUFB6*, *COX6C*, *COX5B*, *BAD*, *COX7A2*, and *NDUFC1* (Figure 4B). As shown in Figure 4C, the dot plots revealed that *APP*, *COX7A1*, and *BAD* were more specifically expressed in endothelial cells and pericytes on FVM of PDR. Hence, *APP*, *COX7A1*, and *BAD* were the major predicted genes that correlated PDR and AD and are also involved in the vasculature. It was also noted that there were 51 and 73 genes that overlapped in three clusters related to PD and HD, respectively (Supplementary Figures S1A, B; Supplementary Tables S6, S7). They both had 12 genes with high expression in the endothelial cell and pericyte clusters, such as *PARK7* and *POLR2L* (Supplementary Figures S1C–F). In addition to the impaired function of the vasculature as the inferred connection between PDR and AD, low-grade inflammation and immune cell activation are also important factors for retinal neurodegeneration. Some pro-inflammatory factors like IL-1β, IL-6, TNF-α, and CCL2 were mainly secreted from the immune cells (microglia, macrophages, dendritic cells, and neutrophils) in the retina tissue (Supplementary Figure S1G). On the other hand, molecules related to adhesion function like intercellular adhesion molecule-1 (ICAM-1), matrix metalloproteinase-2 (MMP-2), and MMP-9 might result in microvascular damage and inflammation by releasing cytokines, which were mainly secreted from the immune cells, endothelial cells, and pericytes in PDR.

### APP gene was widely expressed in the hippocampus vasculature of AD

**FIGURE 5:**
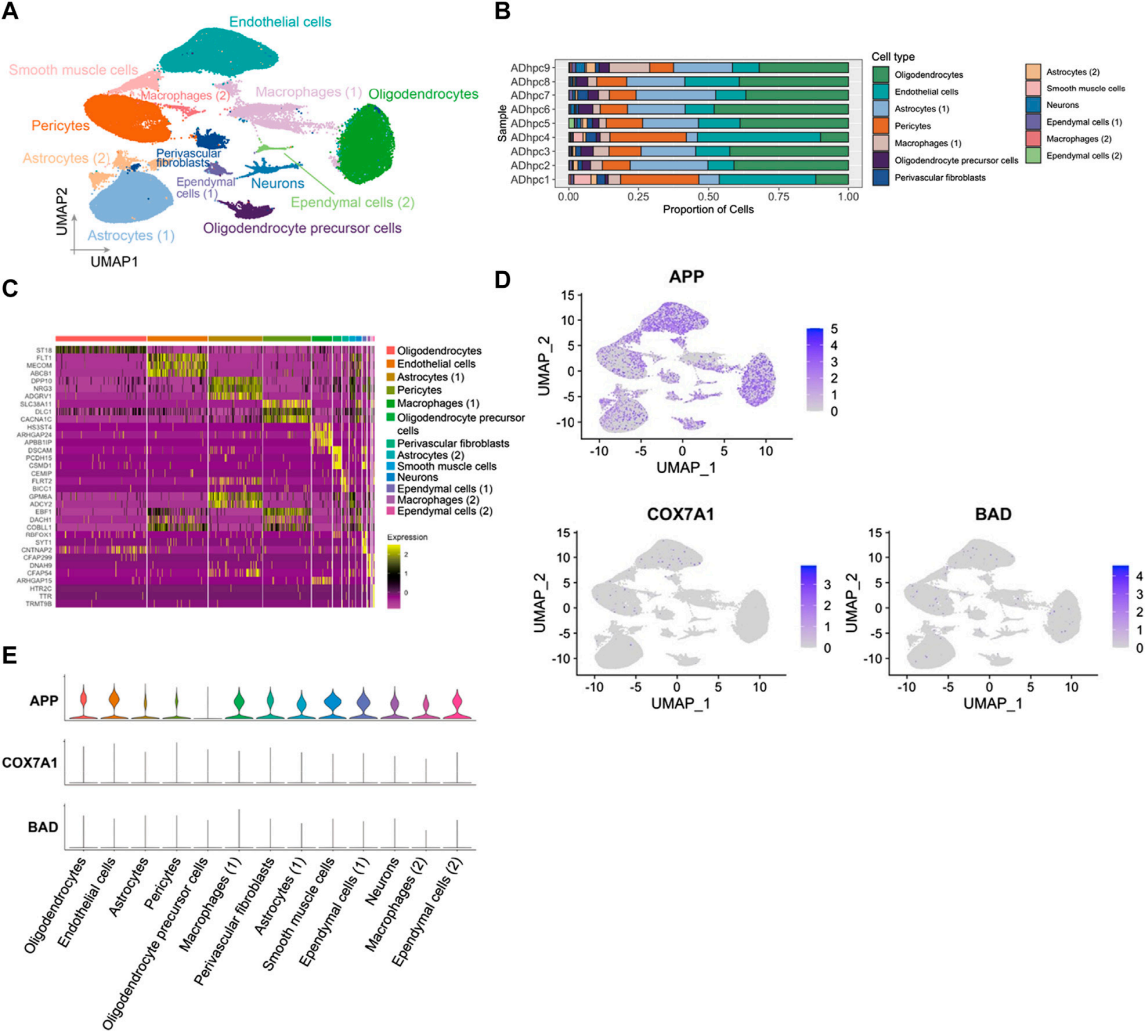
*APP* was widely expressed in various cell types in hippocampus blood vessels of AD. **(A)** UMAP displaying a total of 13 cell clusters in hippocampus vessel samples. **(B)** Bar plot showing the distribution of cell type proportions across hippocampus blood vessel samples from AD patients based on snRNA-seq. Each cell type is represented by a distinct color as shown in panel **(A)**. The number of cells in each cell type per donor is listed in Supplementary Table S8. **(C)** Heatmap of top three marker genes in each cluster. **(D)** Feature plot of *APP*, *COX7A1*, and *BAD* genes in the hippocampus vasculature of AD. **(E)** Expression level of *APP*, *COX7A1*, and *BAD* genes in each cell cluster.

A total of 98,867 nuclei of human hippocampus vessel samples from AD were enriched and separated by UMAP into 13 major cell types (Figures 5A,C). The cell clusters were composed of endothelial cells, pericytes, astrocytes, oligodendrocytes, smooth muscle cells, macrophages, perivascular fibroblasts, ependymal cells, neurons, and oligodendrocyte precursor cells. The distributions of cell type proportions across the hippocampus blood vessel samples showed that endothelial cells and pericytes accounted for over 50% of the total (Figure 5B; Supplementary Table S8). In the analysis of PDR, *APP*, *COX7A1*, and *BAD* were indicated as the key genes involved in the vasculature of PDR and AD. We mapped *APP*, *COX7A1*, and *BAD* genes onto the cell clusters and found that *APP* was expressed by almost all cell types in the vasculature from the hippocampus of AD (Figure 5D). However, *COX7A1* and *BAD* were less expressed by cells in the hippocampus vasculature of AD (Figure 5E). This suggested that *APP* might be the major mediator and perform key functions in the vasculature of both PDR and AD. Our analyses did not identify significant changes in *APP* gene expression in the retina tissue between PDR and the control group in the total RNA transcriptomics study (Supplementary Figure S2). Since FVM is the pathological structure in PDR, the total RNA transcriptomics study of the retina tissue failed to focus on the vasculature as FVM and compare the expression with that of the control samples. Furthermore, we hypothesized that *APP* might interact and function in the complex regulatory networks contributing to the retinal vasculature development instead of direct regulation of expression, and we further investigated the interaction of *APP* with the function of the endothelial cells and pericytes in PDR.

### Endothelial cells and pericytes exhibited a global cellular communication via APP signaling in FVM of PDR

**FIGURE 6:**
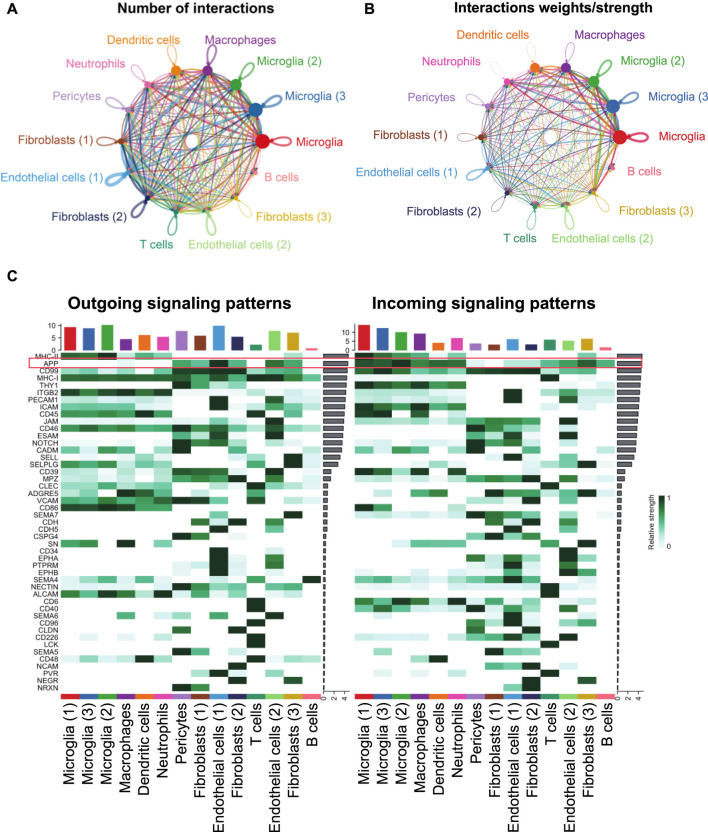
Inferred intercellular communication network revealed the role of APP signaling among cells in PDR. **(A)** Number of interactions. **(B)** Strength of cellular interactions. **(C)** Outgoing and incoming signal strength of each signaling pathway in each cell population in PDR.

The intercellular communication network involves signal transmission among cells, which could reveal the coordination of cells and signals for different biological functions. CellChat predicted how cells posed an influence on other cells or were influenced by other cells. From the scRNA-seq data of PDR, endothelial cells (1) had more paracrine and autocrine interactions than other cell clusters, while three microglial populations had stronger interactions than those of other cells. Specifically, there existed many strong interactions between the two endothelial cell populations (Figure 6A; Supplementary Tables S9, S10). The detected significant ligand–receptor pairs were categorized into 47 signaling pathways among the 14 cell groups, and the signaling role analysis of all signaling pathways showed that microglial, endothelial cell, and pericyte populations were the major signal source, and the microglia were the main signal receiver (Figure 6B). More importantly, APP was one of the leading highly expressed pathways in both signaling source and reception in FVM of PDR (Figure 6C). We further illustrated how APP communicated among cells in FVM of PDR.

**FIGURE 7:**
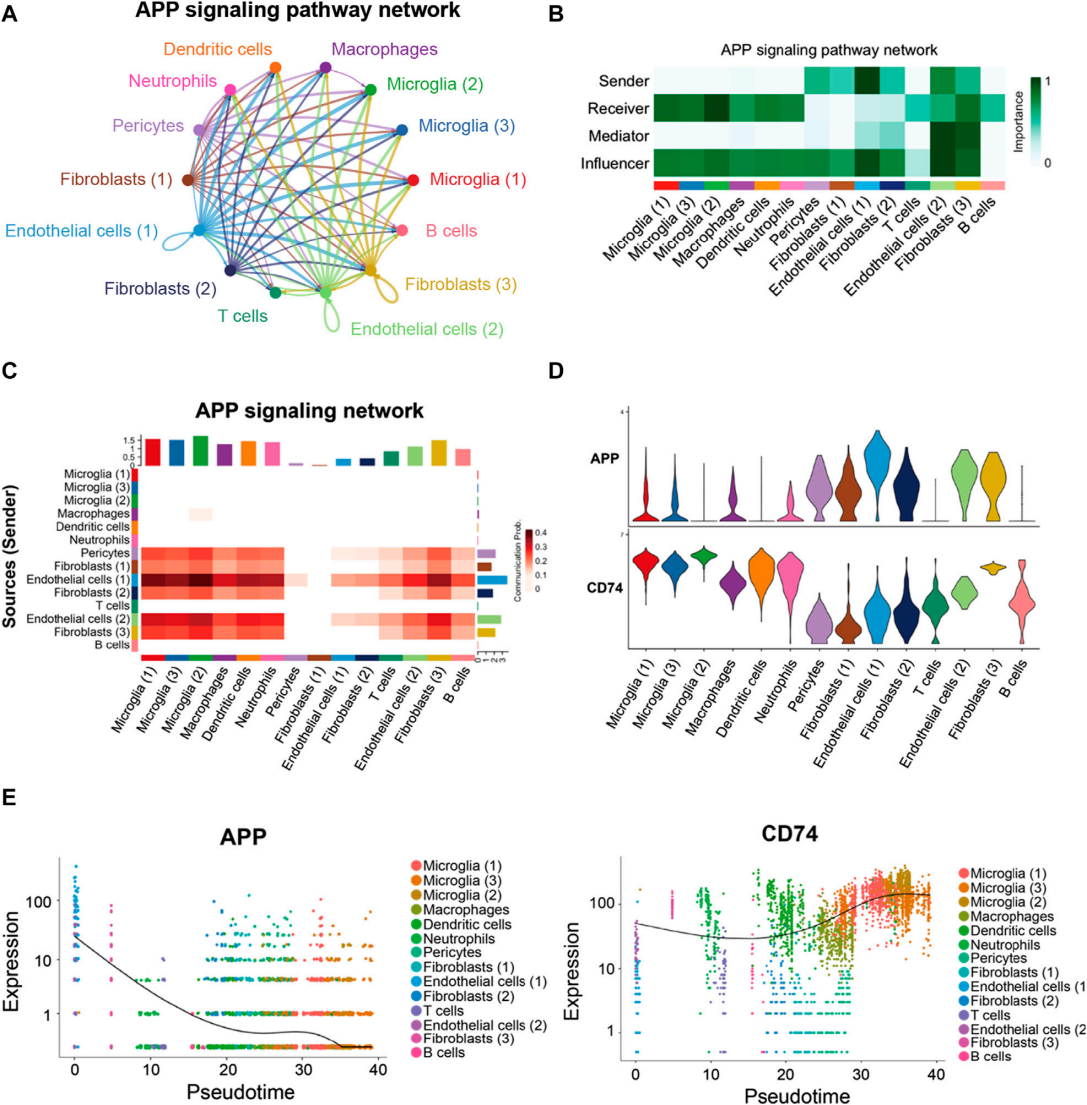
APP signaling pathway inferred by cell–cell communication in PDR. **(A)** Circle plot of the inferred intercellular communication network associated with the APP signaling network in PDR. **(B)** Heatmap showing the relative importance of each cell group in the APP signaling pathway. **(C)** Heatmap of the APP signaling pathway–mediated intercellular communication intensity. **(D)** Expression level of the APP signaling ligand and receptor in each cell population. **(E)** Pseudotime kinetics of the APP signaling ligand and receptor from the root of endothelial cells and pericytes.

As shown in Figure 7A, a majority of the APP interactions among the cells were paracrine, while only endothelial cells and one fibroblast population displayed significant autocrine signaling. Network centrality analysis of the inferred APP signaling (APP-CD74) network revealed that endothelial cells, fibroblasts, and pericytes were the major sources for APP acting onto microglial and other immune cells (Figure 7B). One endothelial cell population is also a prominent mediator in the APP signaling pathway. In addition, APP signaling mediated by APP-CD74 generated from the endothelial cells and pericytes could be received by most population of cells in FVM of PDR (Figure 7C). The ligand *APP* gene was highly expressed by endothelial cells, pericytes, and fibroblasts, while microglial, macrophages, dendritic cells, neutrophils, and one fibroblast population had a high level of receptor *CD74* gene expression (Figure 7D). These results indicated that endothelial cells and pericytes had a global cellular communication via APP signaling in FVM of PDR. The scatter plot shows the dynamic expression of genes with pseudotime values and that the *APP* gene displayed a high level of expressions in the endothelial cells and pericytes at the beginning of PDR, but as the diseases progressed, APP was mainly expressed in the microglia with a low level of expression (Figure 7E). In addition, the expression of CD74 was enhanced in the microglia at the late stage.

### Endothelial cells and macrophages coordinated on APP signaling in the hippocampus blood vessels of AD

**FIGURE 8:**
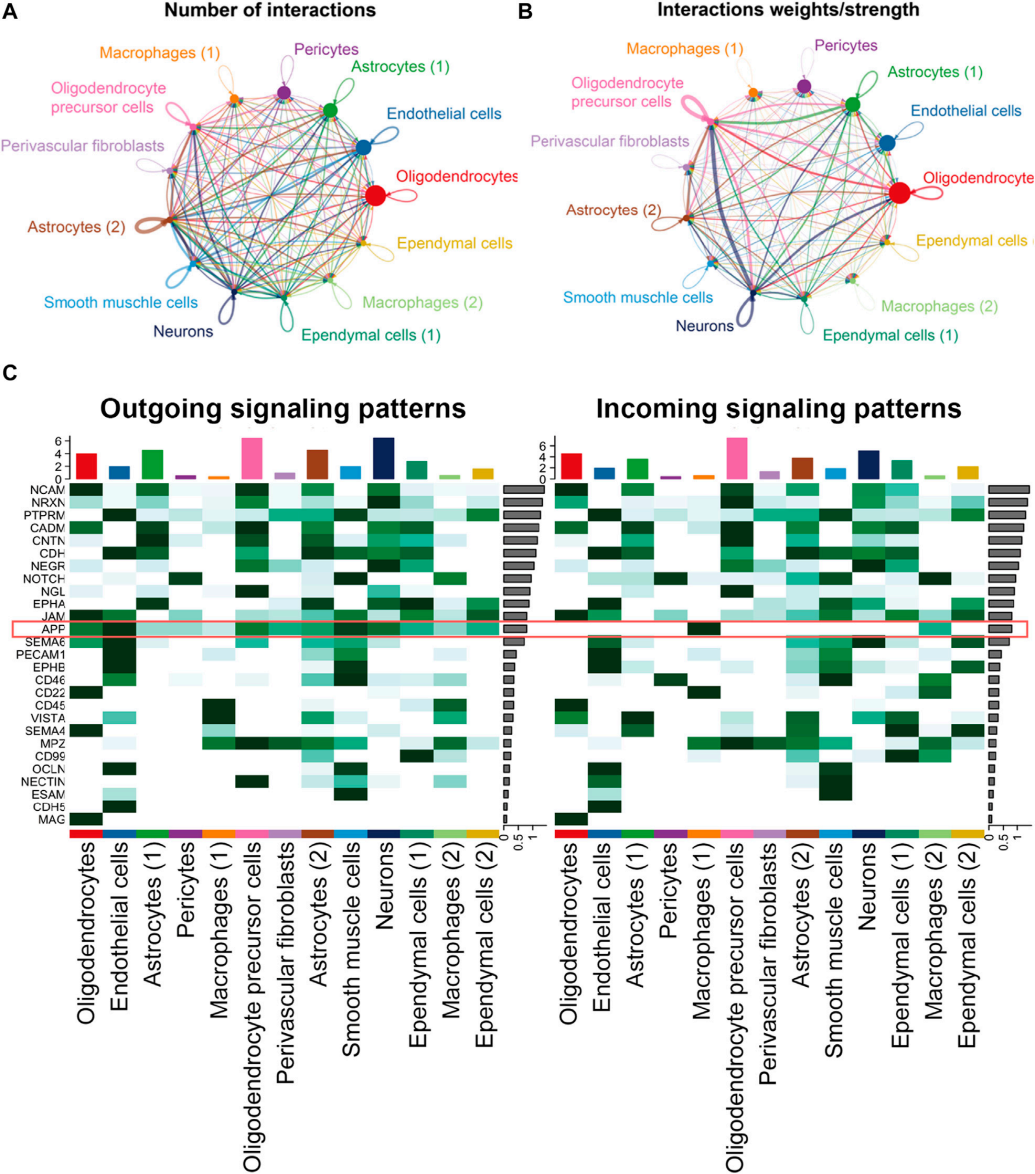
*APP* was one of the significant signaling pathways in the cellular communication in the hippocampus vasculature of AD. **(A)** Number of interactions. **(B)** Strength of cellular interactions. **(C)** Outgoing and incoming signal strength of each signaling pathway in each cell population in AD.

**FIGURE 9:**
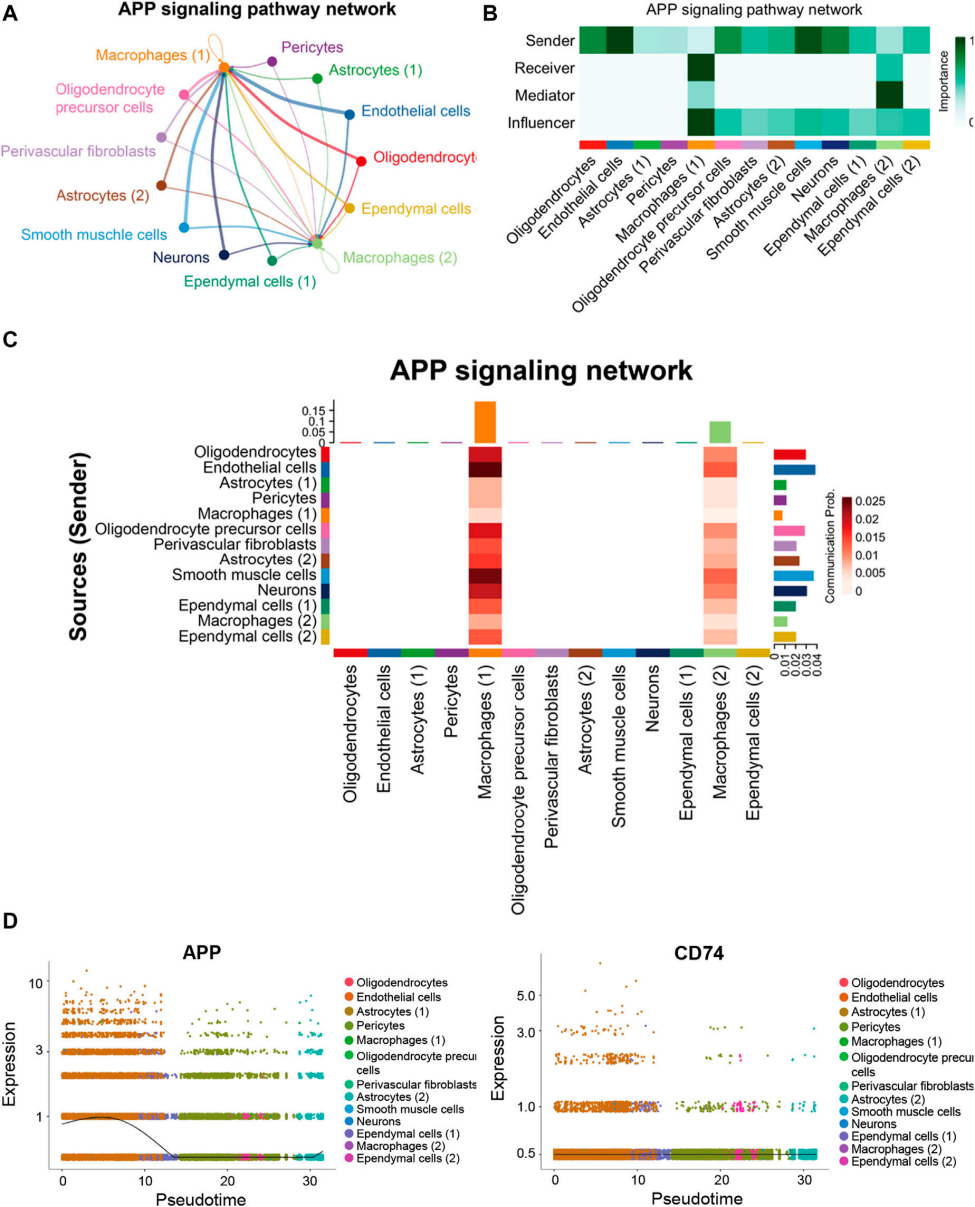
APP signaling pathway inferred by cell–cell communication in AD. **(A)** Heatmap showing the relative importance of each cell group in the APP signaling pathway. **(B)** Circle plot of the inferred intercellular communication network associated with the APP signaling network in AD. **(C)** Heatmap of APP signaling pathway–mediated intercellular communication intensity. **(D)** Pseudotime kinetics of the APP signaling ligand and receptor from the root of endothelial cells.

In the hippocampus blood vessels of AD, astrocytes (2) displayed more autocrine interactions, and oligodendrocyte precursor cells had stronger autocrine interactions (Figures 8A,B; Supplementary Tables S11, S12). In addition, neurons posed a significant influence on the oligodendrocyte precursor cells in the hippocampus of AD. The results revealed that APP signaling was one of the significant signaling pathways in the hippocampus blood vessels of AD (Figure 8C). A majority of cell clusters were the source of APP signaling, and endothelial cells and smooth muscle cells were the leading ones. In addition, macrophages were the prominent receivers, mediators, and influencers, controlling the communications of the APP signaling pathway (Figures 9A,B). It was found that the endothelial cells were the driving sources of APP to macrophages in the hippocampus vasculature of AD (Figure 9C). Furthermore, the scatter plot showed that the APP gene was highly expressed in endothelial cells at the early stage and then the expression was reduced, while the expression of CD74 in AD was little changed due to low expression (Figure 9D). On the other hand, endothelial cells from AD patients also displayed a high outgoing signal strength for several signaling pathways related to inflammation, such as SEMA6, PECAM1, and CD46 (Figure 8C). AD hippocampus vessels produced and secreted inflammatory mediators related to SEMA6 and PECAM1 signaling pathways from the endothelial cells, which also bound to their own receptors to initiate signal transduction. As immune cells, CD46 derived from the endothelial cells influenced the pericytes and macrophages.

### APP gene had a significant correlation with GWAS AD risk genes

**FIGURE 10:**
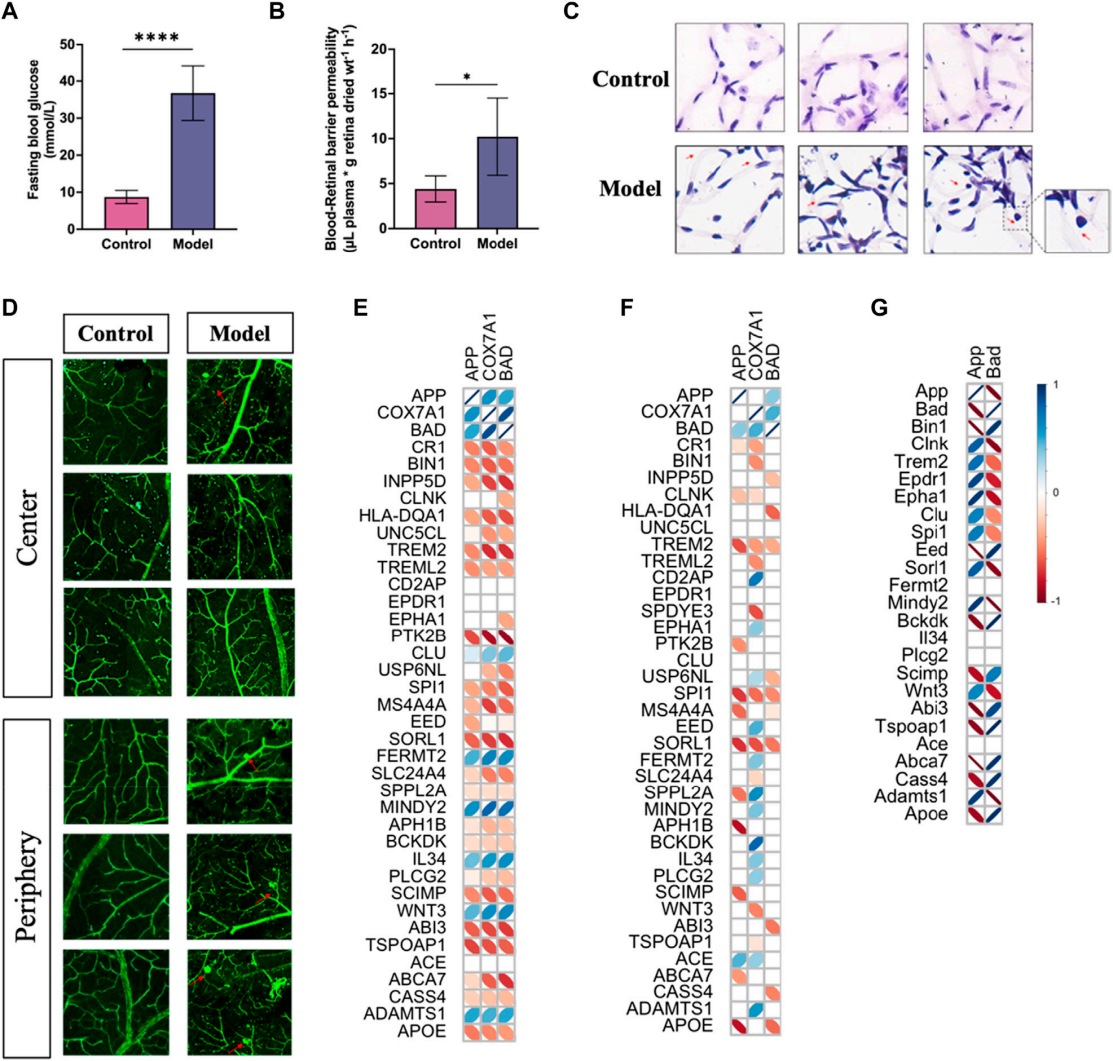
PDR developed in mice with type I diabetes and the correlation of *APP* gene with GWAS AD risk genes. **(A)** Fasting blood glucose levels in control and model groups (^****^ *p* < 0.001). **(B)** Blood–retinal barrier permeability assessed by Evans blue dye. **(C)** H&E staining study of retinal vessels prepared by trypsin digestion. **(D)** Morphology of vessels in the retina tissues by immunofluorescence study. **(E)** Correlation analysis of the *APP* gene with GWAS AD risk genes in PDR scRNA-seq. **(F)** Correlation analysis of the *APP* gene with GWAS AD risk genes in AD snRNA-seq data. **(G)** Correlation analysis of the *APP* gene with GWAS AD risk genes in PDR bulk RNA-seq data. An insignificant correlation (*p* > 0.05) is displayed as the blank box.

To confirm the correlation of predicted gene expressions from PDR with the development of AD, we performed the Pearson correlation analysis on the GWAS genes already known to be associated with the risk of developing AD and related dementias (Bellenguez et al., 2022; Miyashita et al., 2022). Compared to the analysis of PDR and AD, the expressions of *APP*, *COX7A1*, and *BAD* genes displayed a more consistent correlation in PDR than in AD, which may be due to the lower expression of *COX7A1* and *BAD* in the vasculature from the AD hippocampus (Figures 10E,F; Supplementary Tables S13, S14). For validating the role of the *APP* gene in the risk of AD, we developed PDR in a mouse model with STZ-induced T1DM (Figure 10A), in which the blood–retina barrier permeability was enhanced (Figure 10B) and the number of acellular vessels (Figure 10C) and microaneurysms (Figure 10D) increased in the retinal microvasculature. As a result, the *APP* gene had a significant correlation with several GWAS AD risk genes in bulk RNA-seq of the PDR retina (Figure 10G). The expression of the *APP* gene was demonstrated to be negatively correlated with *SCIMP*, *ABI3*, *ABCA7*, and *APOE* and positively associated with *MINDY2* and *ADAMTS1* in the risk of developing AD.

## Discussion

The incidence of PDR has been reported to be associated with the risk of developing AD, and DR has been proposed as a potential biomarker or predictor of cognitive decline and AD in recent years since the retinal and cerebral microvasculature presented similar structures and functions, and their pathologies are both involved with neurovascular regulation (Patton et al., 2005). Hence, there might be underlying interactions between PDR and AD in the vasculature. PDR as the advanced stage of DR is characterized by retinal neovascularization that could damage the blood–retinal barrier, resulting in the formation of FVM (Duh et al., 2017). The endothelial cells and pericytes play an important role in the formation of FVM and maintenance of vascular microenvironment in the retina (Nakao et al., 2013; Qin et al., 2020; Yang et al., 2022). They are the main sources of the vascular endothelial growth factor (VEGF) and connective tissue growth factor (CTGF) and stimulate the formation of FVM (Pi et al., 2011; Kanda et al., 2012). Due to the specific structure of the BBB, the brain vasculature plays an important role in the pathology of AD (Chow and Gu, 2015). The brains of people who suffered and died from advanced AD often have severe damage to the network of blood vessels, and many of the top AD risk genes were found significantly expressed in the blood vessels (Yang et al., 2022). In our study, the endothelial cells and pericytes from FVM of patients with PDR and the hippocampus vessel samples from patients with AD were selected to investigate the underlying interactions between PDR and AD in the vasculature. Moreover, we also found that the retinal vasculature in PDR might have an association with other neurodegenerative diseases like PD and HD. A nationwide cohort study in Korea with 2,362,072 individuals found that the coexistence of T2DM and retinopathy had a stronger association with the risk of having PD than a single risk factor (Han et al., 2023). The progression of PD is mainly involved with the dysfunction and degeneration of dopamine-producing cells in the substantia nigra, and the mechanism underlying the association between PD and PDR has not been elucidated to be relevant with the function of vasculature, while mitochondrial dysfunction, endoplasmic reticulum stress, and inflammation might contribute more to the relationship between metabolic disorders and PD (Santiago and Potashkin, 2013; Rhee et al., 2020; Smajic et al., 2022). HD is a neurodegenerative genetic disorder that is primarily caused by a mutation in the huntingtin gene and affects the function of the central nervous system (CNS), and a single-cell dissection of the human brain vasculature revealed that during the progression of HD, the innate immune signaling was activated in the vascular and glial cell types and the endothelial tight junction protein expression was reduced, which impaired the integrity of the BBB (Garcia et al., 2022).

Our findings illustrated that the *APP* gene as the major mediator in the interactions of PDR and AD was globally and highly expressed in the blood vessels of the AD hippocampus. APP is abundantly found in the membrane and often functions in maintaining the normal proliferation and adhesion of cells. However, the mutations and proteolysis of APP could produce Aβ, which is the primary component of amyloid plaques in the brain of patients with AD, indicating that APP is mainly involved with the processing of Aβ. The imbalance of brain endothelial APP between its production and clearance could lead to Aβ deposition on vessels in the CNS and poses a high risk to individuals of developing cerebrovascular disease and dementia, including AD (Mawuenyega et al., 2010; Ristori et al., 2020). In addition, the retinal vasculature changes in diseases like PDR might be associated with the expression of APP. Retinal capillary degeneration was significantly observed in the retina of transgenic APP/PS1 mouse models of AD that overexpressed the APP protein (Golzan et al., 2017; Shi et al., 2020). A study also demonstrated that diabetes-induced retinal neurodegeneration and brain neurodegeneration shared several common pathogenic pathways, and “amyloid processing” was one of the main pathways (Sundstrom et al., 2018). In addition to APP, other molecules like pro-inflammatory factors might be secreted from retinal tissues to be involved in neurodegeneration. Our findings demonstrated that some pro-inflammatory factors were primarily derived from the immune cells in retinal tissues. These findings are aligned with reports indicating that the activated microglia, resident immune cells of the retina, and infiltrating monocytes are crucial for the regulation the diabetes-induced inflammation. The pro-inflammatory factors contribute to the impairment of the blood–retina barrier and neurovascular unit and damage in neurons (Simó et al., 2018). Neuroinflammation has a prominent role in the pathogenesis of AD (Leng and Edison, 2021). Sema6D has been demonstrated to promote the phagocytic uptake of oligomeric Aβ and the release of inflammatory cytokines like IL-1β, IL-6, TNF-α, and IL-8, regulating the function of microglial cells to increase the risk of AD (Finan et al., 2022). Additionally, CD46 is expressed in endothelial cells and glial cells in the human brain, and it could mediate inflammatory response and phagocytic activity, promoting the microglial Aβ clearance, while dysfunction of these processes might contribute to the accumulation of Aβ and neuroinflammation in the pathogenesis of AD (Salminen et al., 2013).

The ligand–receptor interactions performed in the scRNA-seq data contribute to unveiling the signaling pathways of intercellular communications. In the data set of PDR, the cellular communication analysis revealed that endothelial cells had more paracrine and autocrine interactions than the other cells, and two endothelial cell populations had strong interactions. It is consistent with the neovascularization in PDR, which might drive vascular cells to be the major source of signaling to influence other cells. Our study revealed that the APP signaling pathway is primary in cells on FVM of PDR, and APP-CD74 is the leading ligand–receptor contributor to this pathway. As the membrane protein in endothelial cells, APP maintains the normal endothelial functions and responses to VEGFA (Ristori et al., 2020). In addition, angiogenesis and neurodegeneration in retinal diseases could be attributed to APP-induced Aβ and neuroinflammation (Anderson et al., 2008; Ning et al., 2008; Cunvong and Cameron, 2013; Salobrar-Garcia et al., 2021). CD74 is a transmembrane protein of the cell surface receptor for macrophage migration inhibitory factor (MIF) with immune response (Zheng et al., 2012; Gil-Yarom et al., 2017). The level of CD74 was increased in the intraocular microenvironment of patients with PDR, especially those with significant active angiogenesis (Abu El-Asrar et al., 2019). The pseudotime analysis of PDR indicated that endothelial cells and pericytes first had a high level of *APP* expression to influence other cells in PDR and it later expanded to the major cell populations and triggered other molecules like its receptor CD74 during the progress of PDR. Overall, the angiogenesis of PDR was indicated to suggest the influence of vascular cells on other cells via the APP-CD74 pathway, which might also increase the risk of neurodegeneration.

The cellular communication results show that APP-CD74 signaling was one of the major signaling pathways in the blood vessels from the hippocampus of AD. The research has proven that CD74 could interact with APP to inhibit the production of Aβ, and CD74 changed the intracellular distribution of APP and disrupted normal trafficking of APP in neurons instead of reducing the secretion of sAPPα or sAPPβ, and thus, the APP-CD74 signaling pathway was crucial for AD pathology (Matsuda et al., 2009; Kiyota et al., 2015). Additionally, endothelial cells were found to be the leading source of APP signaling, and the macrophages were the major receiver, mediator, and influence of this signaling. It was consistent with the reported results that APP could mediate the production of Aβ and affect the density of brain perivascular macrophages in the vasculature of the hippocampus (Karam et al., 2022). A previous study found that the recruited macrophages could enhance the vascular Aβ phagocytosis for immunotherapy in APP/PS1 mice (Zuo et al., 2021). In addition, the deposition of vascular Aβ in the cerebral vasculature could cause breakdown of the vascular integrity and a decrease in the number of cerebrovascular endothelial cells and pericytes (Sweeney et al., 2018). Our findings further validated that *APP* gene expression presented a significant correlation with GWAS AD risk genes in both PDR and AD data sets. Overall, the APP signaling pathway played an important role in AD pathology and regulating vascular functions in PDR, and it provided a novel target for investigating the vasculature interactions in PDR and AD.

For the limitations, a large sample size is recommended to fully reflect the changes in these single cells in the pathologies of PDR and AD. Additional *in vitro* and *in vivo* experiments are required to further elucidate how the dysfunction of APP in retinal vasculature leads to the activation of APP expression in cerebral vasculature and the accumulation of Aβ. Further clinical studies should evaluate the mutations of APP and its mediated pathways or molecules in both PDR and AD patients, and this could be implemented in PDR screening programs to reduce the risk of developing progressive neurodegenerative diseases like AD.

## Conclusion

The present study demonstrated that the APP signaling pathway is of great importance in the interactions of vasculature between PDR and AD by analyzing the scRNA-seq data of human samples. Endothelial cells and pericytes were found to pose an influence on other cells in PDR via the APP signaling pathway. The endothelial cells in the hippocampus of AD coordinated with macrophages in the vasculature via the APP signaling pathway. The expression of the *APP* gene had a significant correlation with that of AD risk genes. Our study revealed the role of the APP signaling pathway in the vasculature of PDR and AD, and it provided a potential target for exploring the interactions in vasculature development of PDR and AD and predicting the risk of developing AD in PDR patients.

## Data Availability

The original contributions presented in the study are included in the article/Supplementary Material; further inquiries can be directed to the corresponding author.
