# Supplementary material for: Single-cell transcriptome profiling highlights the role of APP in blood vessels in assessing the risk of patients with proliferative diabetic retinopathy developing Alzheimer’s disease
Source: Front Cell Dev Biol. 2024 Jan 24;11:1328979. doi: 10.3389/fcell.2023.1328979 (PMC10847282; doi:10.3389/fcell.2023.1328979)
Supplement: Supplementary file 13 [file DataSheet1.docx]

**Figure legends**

**Figure S1.** **Identification of genes from endothelial cells and pericytes in PDR related to PD and HD pathway.**

**a**, Integration of KEGG enrichment genes in PD pathway from endothelial cells and pericytes in PDR.

**b**, Integration of KEGG enrichment genes in HD pathway from endothelial cells and pericytes in PDR.

**c,** Integration of highly expressed genes in both endothelial cells and pericytes with PD pathway-related genes. PD related genes: 51 overlapping genes in **a**.

**d,** Integration of highly expressed genes in both endothelial cells and pericytes with HD pathway-related genes. HD related genes: 73 overlapping genes in **b**.

**e,** Feature plot of 12 PD-related genes with high expression in endothelial cells and pericytes clusters.

**f,** Feature plot of 12 PD-related genes with high expression in endothelial cells and pericytes clusters.

**g,** Feature plot of several proinflammatory factors in endothelial cells and pericytes clusters.

**Figure S2. The gene expression of APP in the retina tissue.**

Volcano and box plots showing gene expression differences of APP in the retina tissues between control and PDR group. Difference was calculated by one-sided Wilcoxon rank-sum test.

**Figures**


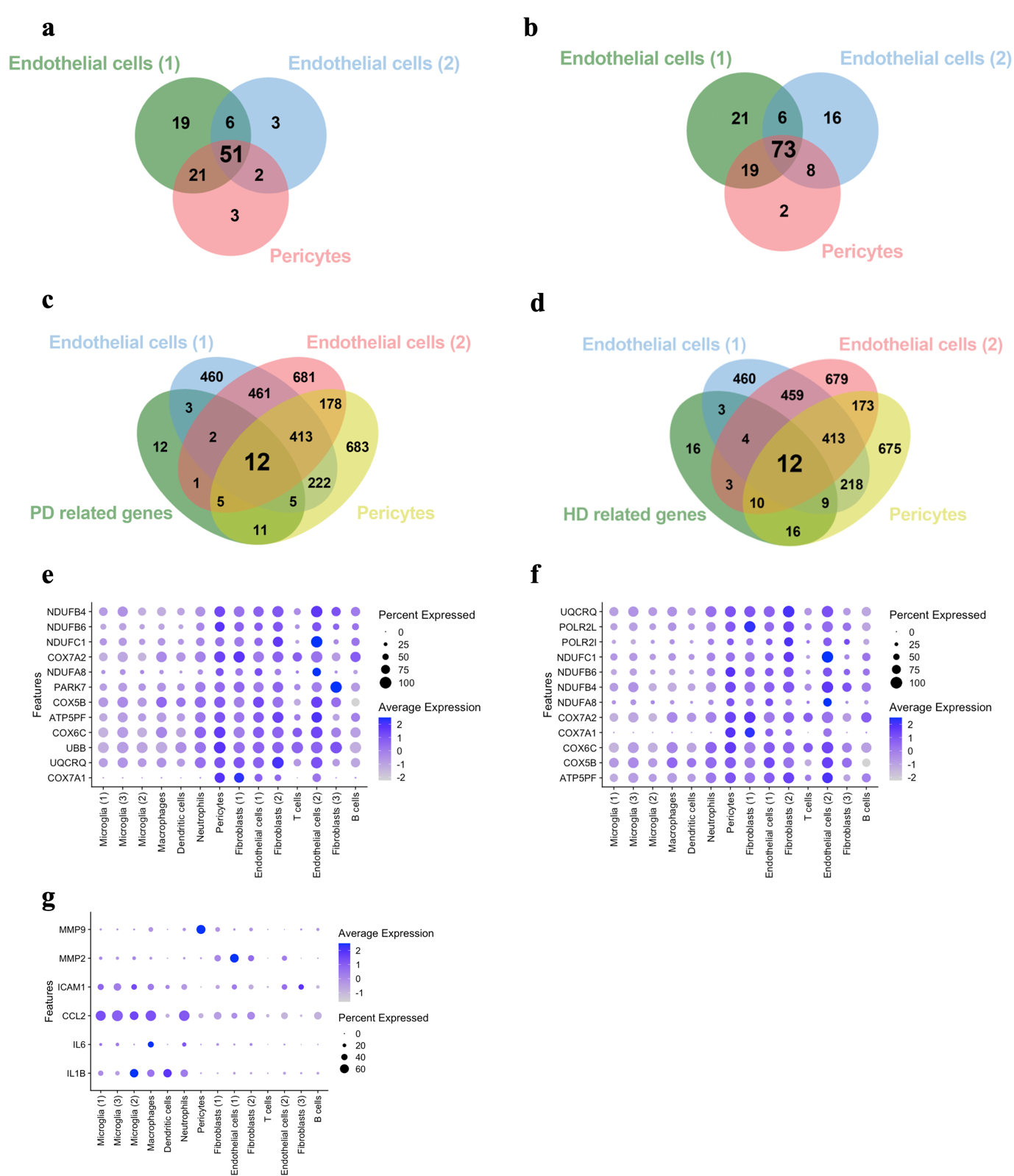


**Figure S1.** **Identification of genes from endothelial cells and pericytes in PDR related to PD and HD pathway.** **a**, Integration of KEGG enrichment genes in PD pathway from endothelial cells and pericytes in PDR. **b**, Integration of KEGG enrichment genes in HD pathway from endothelial cells and pericytes in PDR. **c,** Integration of highly expressed genes in both endothelial cells and pericytes with PD pathway-related genes. PD related genes: 51 overlapping genes in **a**. **d,** Integration of highly expressed genes in both endothelial cells and pericytes with HD pathway-related genes. HD related genes: 73 overlapping genes in **b**. **e,** Feature plot of 12 PD-related genes with high expression in endothelial cells and pericytes clusters. **f,** Feature plot of 12 PD-related genes with high expression in endothelial cells and pericytes clusters. **g,** Feature plot of several proinflammatory factors in endothelial cells and pericytes clusters.

**
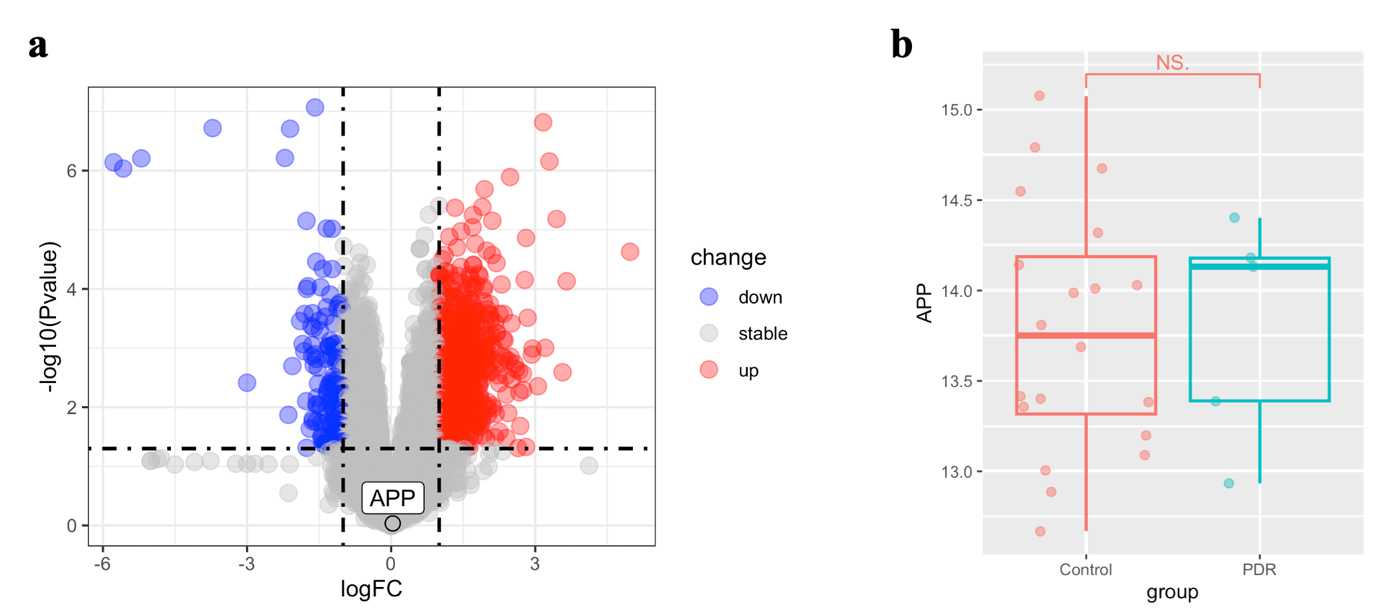
**

**Figure S2. The gene expression of APP in the retina tissue.** Volcano and box plots showing gene expression differences of APP in the retina tissues between control and PDR group. Difference was calculated by one-sided Wilcoxon rank-sum test.
